# COMPARING GAINS AND LOSSES: EXAMINING THE EFFECTS OF INCENTIVES ON WORKING MEMORY CAPACITY IN OLDER ADULTS

**DOI:** 10.1093/geroni/igae098.4143

**Published:** 2024-12-31

**Authors:** Julie Pham, Holly Bowen

**Affiliations:** Southern Methodist University, Dallas, Texas, United States; Southern Methodist Univeristy, Dallas, Texas, United States

## Abstract

Aging is associated with declines in cognition, particularly in areas of working memory responsible for the temporary storage, processing, and manipulation of information. Prior studies have shown that the use of incentives can be successful at modulating cognition in older age. However, much of the previous work has largely focused on reward incentives. Limited research on loss avoidance incentives has been done to understand whether its use can differentially improve cognition, especially in working memory capacity. Arguably, this is an important area of focus as older adults can experience greater losses with age, such as in health, independence, and social networks, that may shift decision-making priorities towards conserving resources and minimizing decline. The current study aimed to investigate whether older adults prioritize gains or losses differently while completing a spatial working memory task. An online sample of 75 older adults (ages 60+) were recruited using the Cloud Research Connect Platform. Each participant completed both a reward and loss avoidance block using a single-item change detection task discriminating between different colored squares. A paired samples t-test was conducted to evaluate the effect of reward and loss avoidance conditions. Findings did not indicate a significant difference in working memory performance between conditions. This suggests that use of either rewards or loss incentives may not differentially improve working memory capacity and can both have potential benefits in modulating cognitive function in an older adult sample.

